# An Interdisciplinary Approach for Rehabilitating a Patient with Amelogenesis Imperfecta: A Case Report

**DOI:** 10.1155/2012/432108

**Published:** 2012-08-16

**Authors:** Niloufar Khodaeian, Mahmoud Sabouhi, Ebrahim Ataei

**Affiliations:** ^1^Dental Implant Research Center and Department of Prosthodontics, School of Dentistry, Isfahan University of Medical Sciences, Isfahan, Iran; ^2^Torabinejad Dental Research Center, Department of Prosthodontics, School of Dentistry, Isfahan University of Medical Sciences, Isfahan 81746-73461, Iran; ^3^Department of Restorative Dentistry, School of Dentistry, Shahid Sadoughi University of Medical Sciences, Yazd, Iran

## Abstract

Amelogenesis imperfecta (AI) has been defined as a group of hereditary enamel defects. It can be characterized by enamel hypoplasia, hypomaturation, or hypocalcification of the teeth. AI may be associated with some other dental and skeletal developmental defects. Restoration for patients with this condition should be oriented toward the functional and esthetic rehabilitation. This clinical report describes the oral rehabilitation of a young patient diagnosed with the hypoplastic type of AI in posterior teeth and hypomatured type of AI in anterior teeth.

## 1. Introduction

Amelogenesis imperfecta (AI) is a diverse group of hereditary disorders that primarily affect the quantity, structure, and composition of enamel [1]. The inheritance pattern of AI may be autosomal dominant, autosomal recessive, or X-linked [2]. According to the Witkop classification system, there are four main forms of AI: type I hypoplastic enamel, type II hypomatured enamel, type III hypocalcified enamel, and type IV hypomatured-hypoplastic enamel with taurodontism [1]. Clinical presentation of AI varies considerably among the different AI types. In the hypomature type, the affected teeth exhibit mottled, opaque white-brown or yellow discolored enamel, which is softer than normal. The hypocalcified type shows pigmented, softened, and easily detachable enamel. In the hypoplastic type, the enamel is well mineralized but its amount is reduced. Clinically, grooves and pits will be realized on the surface of the fine enamel. The tooth is tapered towards the incisal/occlusal face and has open contact points [3, 4]. AI may be associated with some other dental and skeletal developmental defects or abnormalities, such as unerupted teeth, congenitally missing teeth, taurodontism, pulpal calcification, crown and root resorption, cementum deposition, truncated roots, interradicular dentinal dysplasia, gingival hyperplasia, follicular hyperplasia, constricted maxillary arch (omega-shaped arch), reversed curve of Spee, vertical growth pattern, and dental and skeletal open bite [5–17]. The main clinical problems present in AI patients are tooth sensitivity, unsatisfactory esthetics, and loss of occlusal vertical dimension due to the rapid wearing of dentition [18, 19]. Treatment planning for patients with AI is related to many factors: the age and socioeconomic status of the patient, the type and severity of the disorder, and the intraoral situation. An interdisciplinary approach is necessary to evaluate, diagnose, and resolve AI patient problems using a combination of orthodontic, periodontal, prosthodontics, and restorative treatment [3, 20–22]. This clinical report describes the interdisciplinary approach for rehabilitating a patient with AI.

## 2. Case Report

**Figure 1:**
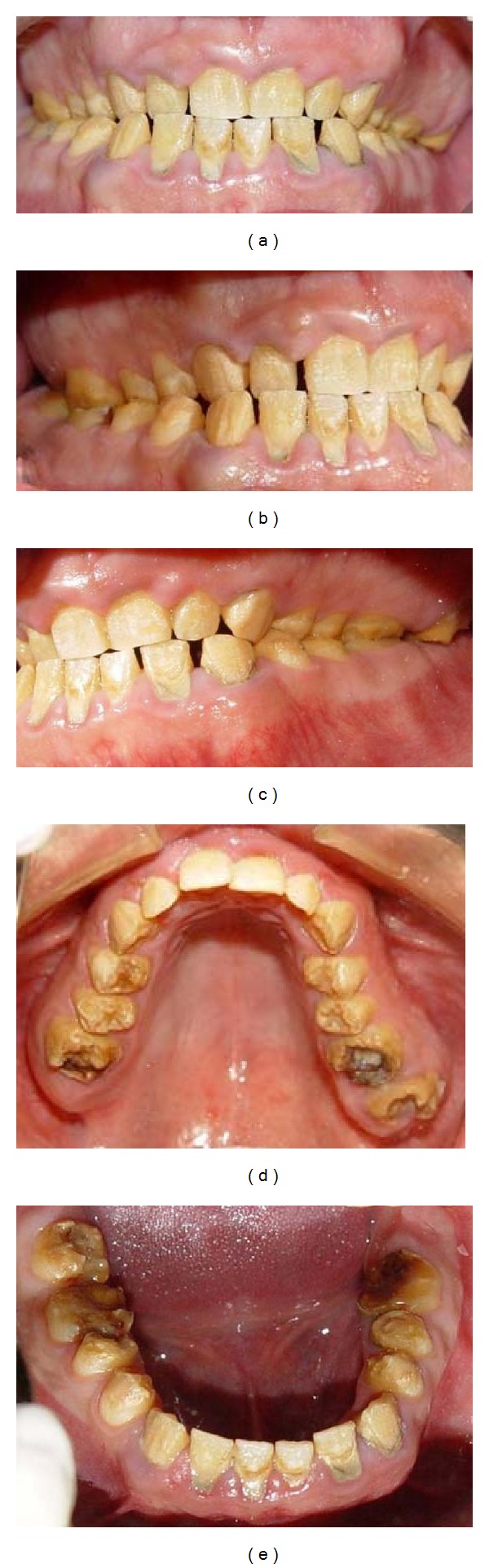
Pretreatment intraoral view: (a) frontal view; (b) right lateral view; (c) left lateral view; (d) maxillary occlusal view; (e) mandibular occlusal view.

**Figure 2:**
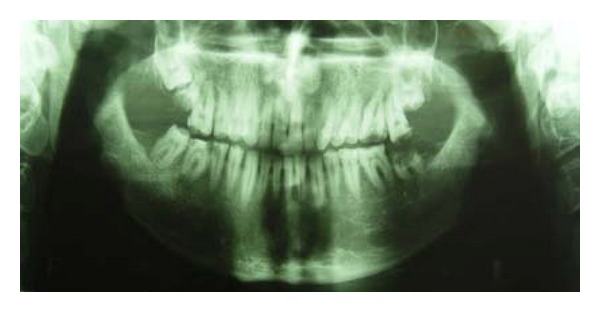
Pretreatment panoramic view.

A 21-year-old male patient presented with a chief complaint of discolored teeth. He did not complain about tooth sensitivity and chewing inability. No remarkable findings were identified in his medical records. Among his first-degree family members, only one of his 2 sisters and his brother exhibited the same dental problems and his parents were cousins, so his inheritance pattern of AI was autosomal recessive. He had not received any prior dental treatment. In extraoral examination, the patient had competent lips and showed no facial asymmetry, no muscle tenderness or palpable lymph nodes, and no signs and symptoms of joint disorder. Intraoral examination revealed hypoplastic and hypomature types of AI in posterior and anterior teeth, respectively. The incisal edges were thin and the cuspal structures were aberrant (Figure 1). There were short clinical crowns, especially in the posterior regions. Other findings included posterior teeth wear and caries on teeth nos. 14, 15, 29, and 30. The patient had constricted maxillary arch and crossbite relationship in bilateral posterior regions and teeth nos. 6 and 7. The patient suffers from pain on teeth nos. 14 and 29. The patient's oral hygiene was generally poor, as evidenced by bleeding on probing and plaque index findings. No packet deeper than 4 mm was recorded and the gingiva was hyperemic and edematous. Esthetic evaluation revealed a “gummy smile” in anterior maxilla and asymmetry in gingival contours of anterior teeth. The interocclusal distance measured at the premolar region during physiological rest was 3 mm. It was not possible to determine the molar relationship, but a Class I canine relationship was observed. The panoramic radiograph shown in Figure 2 revealed a thin layer of enamel along the top of most erupted teeth. The crown of teeth nos. 2 and 18 were resorbed. The teeth nos. 1, 2, 16, and 18 were impacted and the teeth nos. 17 and 32 were missed. The root of all teeth appeared to be normal in size and shape. Several closely approximated roots were also evident. Pulpal involvement was found on teeth nos. 14, 29, and 30.

## 3. Treatment Planning

A treatment plan was developed with the aims of pain control for teeth nos. 14 and 29, generally preventive care and improvement in oral hygiene, caries removal and root canal therapy for teeth nos. 14, 29, and 30, extraction of teeth nos. 1, 2, 15, 16, and 18, orthodontic treatment for managing the anterior and posterior crossbite, periodontal correction of gingival contours in anterior sextants and crown lengthening in posterior sextants, and prosthodontic treatment plan that includes porcelain laminate veneer for maxillary and mandibular incisors and metal-ceramic restoration for other teeth.

## 4. Treatment

**Figure 3:**
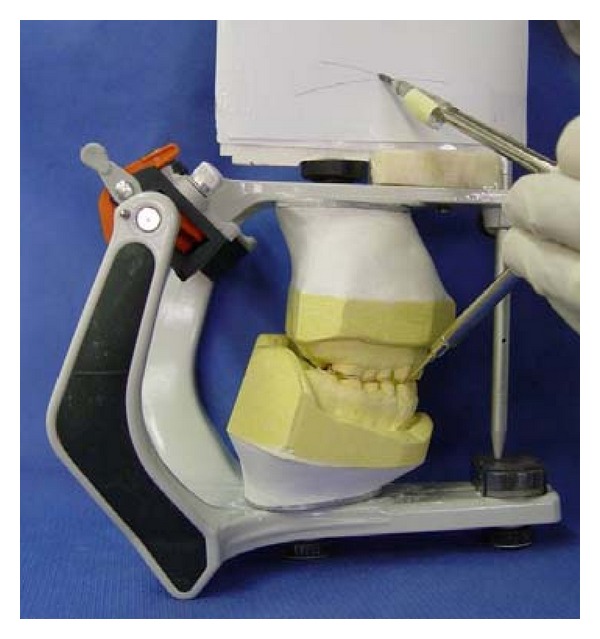
Occlusal plan evaluation using the Broadrick occlusal plane analyzer.

**Figure 4:**
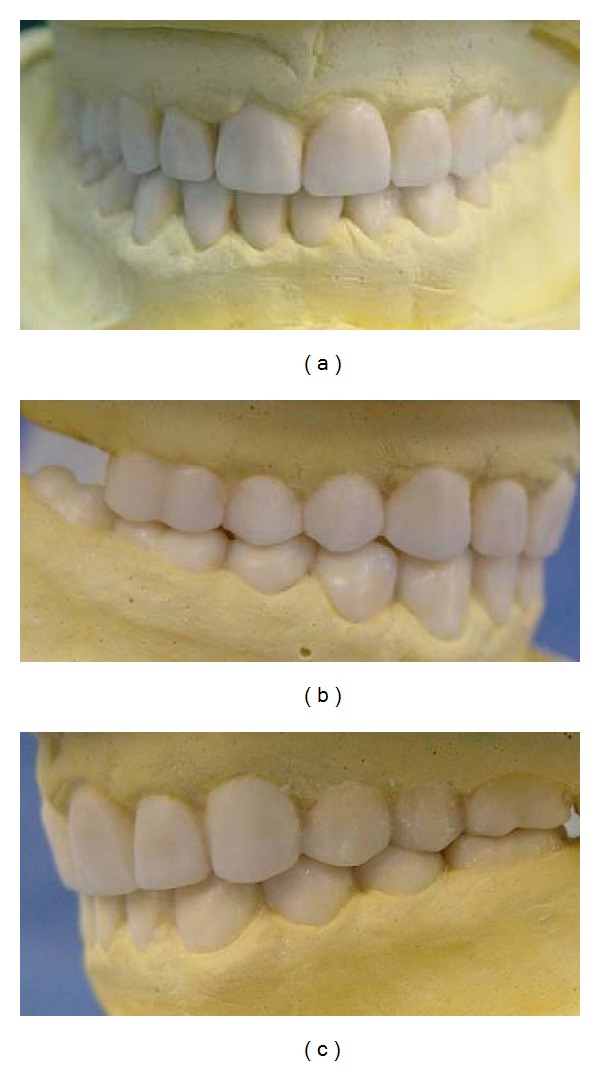
Diagnostic wax-up on diagnostic casts. (a) Frontal view; (b) right lateral view; (c) left lateral view.

**Figure 5:**
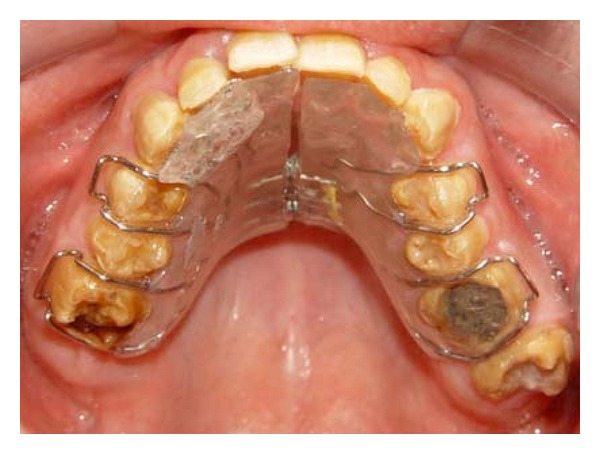
Orthodontic treatment with removable appliance.

**Figure 6:**
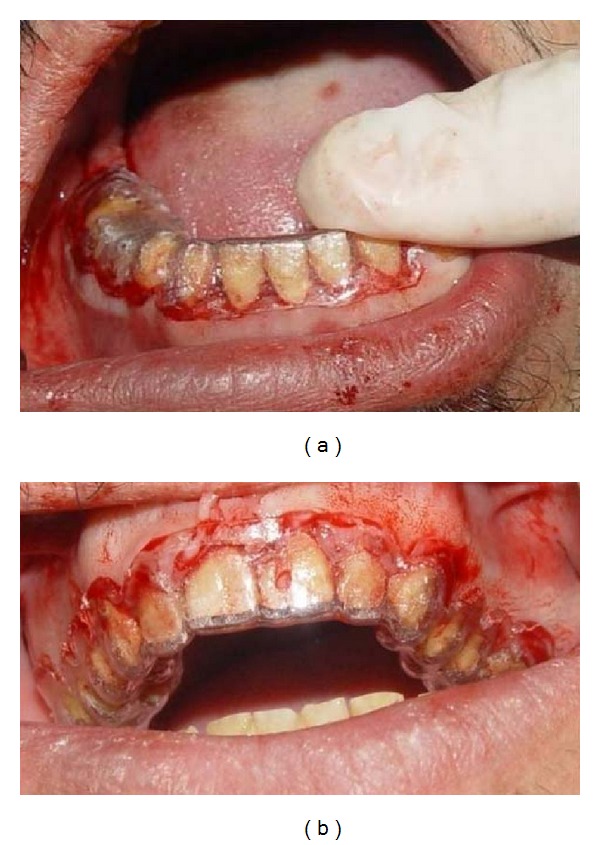
Crown lengthening according to fabricated stent: (a) in mandible; (b) in maxilla.

**Figure 7:**
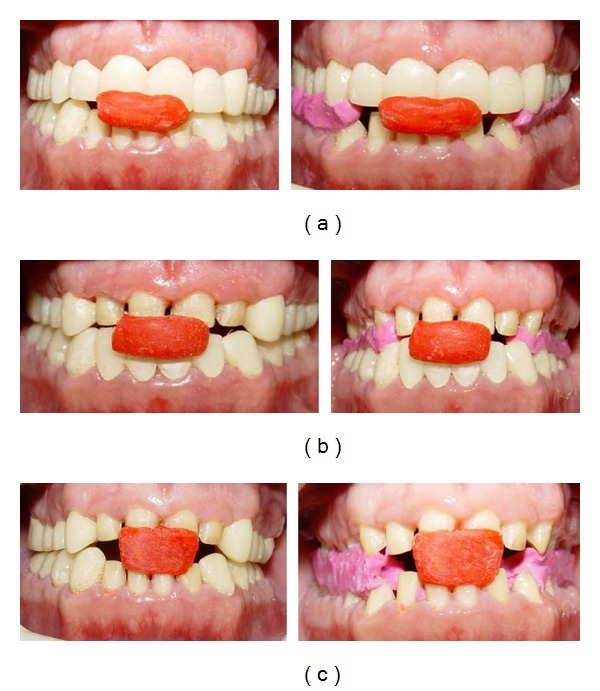
Records for cross-mounting: (a) record of maxillary provisional restorations in relation to mandibular prepared teeth; (b) record of maxillary prepared teeth in relation to mandibular provisional restorations; (c) record of maxillary prepared teeth in relation to mandibular prepared teeth.

**Figure 8:**
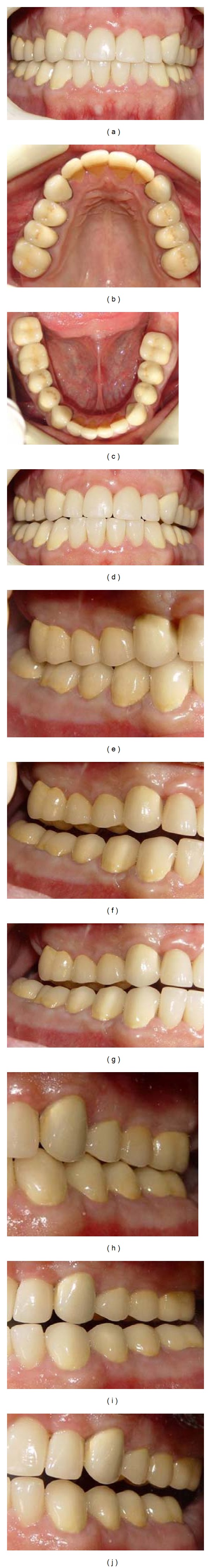
A 1-year posttreatment view of restoration: (a) frontal CR view; (b) maxillary occlusal view; (c) mandibular occlusal view; (d) frontal protrusive view; (e) right-side CR view; (f) right-side nonworking view; (g) right-side working view; (h) left-side CR view; (i) left-side working view; (j) left-side nonworking view.

**Figure 9:**
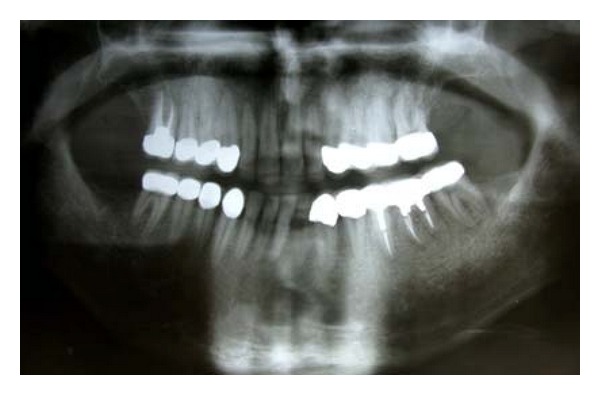
Posttreatment panoramic view.

Maxillary and mandibular arch primary impressions were obtained using a heavy- and light-body vinyl polysiloxane impression material (Panasil, Kettenbach Dental, Eschenburg, Germany). Two sets of diagnostic casts were made by pouring the impressions twice with a type III dental stone (Fuji Rock, GC Dental Corp., Tokyo, Japan). One cast set was used for the diagnostic wax-up and the other was saved for patient records. A centric relation record was taken according to Dawson's bimanual technique [23] by using anterior deprogrammer. The casts were mounted on a semiadjustable articulator (A_7_ Plus articulator, Bio-art, Brazil) using facebow transfer (Professional facebow, Bio-art) and centric relation record. Then, the articulator was adjusted based on excursive records. A Broadrick occlusal plane analyzer showed correct present occlusal plane and curve of spee [24] (Figure 3). The diagnostic wax-up was made according to optimum esthetic and function (Figure 4). After scaling and oral hygiene instruction, the patient adherence to recommended oral health care program was demonstrated after 2 weeks. The gingival edema was resolved and hyperemic appearance of gingival turned to normal. Also, bleeding on probing and plaque index findings became normal. Endodontic treatment for teeth nos. 14, 29, and 30, and extraction of teeth nos. 1, 2, 15, 16, and 18 were done. Amalgam core build up was done on endodontically treated teeth because of adequate coronal structures. Orthodontic treatment using removable appliance lasted about 6 months in active phase and 6 months in passive phase (Figure 5). After completing of orthodontic treatment, periodontal surgery procedures based on fabricated stents were conducted in 4 sessions (Figure 6). After 6 weeks, maxillary and mandibular incisors were prepared for porcelain laminate veneers and other teeth were prepared for metal-ceramic crowns. The amount of reduction was guided by the silicone index of the diagnostic wax-up. Provisional composite veneers (Protemp II, 3 M ESPE, Seefeld, Germany) were fabricated using silicone index for incisors, and laboratory-processed provisional crowns, which were fabricated with the aid of the diagnostic wax-up, were relined (Tempron, GC Dental Products Corp.) for other teeth. The occlusion was adjusted on the provisional restorations to establish a mutually protected scheme in the mouth. Maxillary and mandibular impressions were obtained in vinylpolysiloxane impression material (Panasil, Kettenbach), and irreversible hydrocolloid impressions (Jeltrate, Alginate, Fast set, Dentsply Intl, York, PA, USA) were made to make the cast of temporary restorations after 3 months of periodontal surgery. Cross-mount records were taken to use for mounting of definitive casts and casts of temporary restorations in the same articulator to serve as a guide for the fabrication of definitive restorations (Figure 7). The metal-ceramic restorations (Ivoclar, Vivadent, Schaan, Liechtenstein), and all-ceramic veneers (IPS Empress II, Vivadent) were evaluated intraorally. Then, veneers were etched with 4.9% hydrofluoric acid (IPS Ceramic gel, Vivadent) for 20 seconds and silanized (Monobond-S, Vivadent). Enamel surfaces of incisors were etched with 37% phosphoric acid (Total etch, Vivadent) for 40 seconds, and the veneers were bonded with a light-cured resin cement (Choice2 veneer cement, Bisco, Inc., Schaumburg, USA). The metal-ceramic restorations were cemented with glass ionomer cement (Ketac Cem, 3 M ESPE). The mutually protected occlusion scheme was preserved for this patient to allow for relatively even distribution and less stress of forces during excursive movement. Follow-up visits were scheduled at 3 months and then at 6 months (Figures 8 and 9). No esthetic and functional problems were seen after the 3 years of follow-up period.

## 5. Discussion

The marked phenotypic diversity of autosomal recessive AI cases with a hypoplastic phenotype did not permit straightforward classification according to a currently accepted nosology [25]; this patient had hypoplastic and hypomature types of AI in posterior and anterior teeth. Some of dental and skeletal developmental abnormalities in AI patients were also presented in this case. Crown resorption of unerupted teeth in autosomal recessive AI was already investigated [10]. This case showed preeruptive crown resorption on teeth nos. 2 and 18. Edematous and hyperemic gingiva, several closely approximated roots, and omega-shaped maxillary arch, were the other common abnormalities in AI patients which found in this case. Also, the teeth nos. 1, 2, 16, and 18 were impacted. Seow showed that people with AI have 6 times the tendency of unaffected people to have impaction of permanent teeth [26].

Several clinical reports have been presented describing the restorations of individuals affected by AI [22, 27–32]. Several authors prefer full porcelain restorations as the treatment modality of patient with AI [22, 27–31]. Advances in the field of esthetic dentistry, especially in bonding to dentin, help practitioners to restore function and esthetics to an acceptable level [27]. Nevertheless, marginal adaptation and bonding problems have been pointed out as disadvantages of laminate veneers [22]. Several factors may influence the outcome of restorative treatments including acid-etching and bonding of teeth affected by AI. For example, etch pattern of clinically variants of AI enamel may be altered and not produce a good match to normal enamel [33]. Additionally, the morphological pattern of dentin in hypocalcified AI is relatively similar to sclerotic dentin, which responds to acid etch conditioning differently than normal dentin [34, 35]. Newer dentin-bonding systems provide more reliable bonding to dentin and infiltrate more effectively to enamel prism than did the earlier systems. So, they may provide more durable dentin bonding than traditional methods of bonding to abnormal enamel. Despite severe enamel abnormalities, successful bonding of porcelain restorations could be achieved, and there were few adhesion complications in present case and several previously reported cases [22, 24, 29, 30]. The decision as to whether to preserve an enamel layer and use adhesive restorations or to completely remove the enamel and use complete coverage crowns depends on the extension and depth of the patient's enamel lesions [31]. In the present case, it was determined that the enamel should be presented based upon observation of clinically normal enamel during preparation of incisors. But canines and posterior teeth need full coverage restorations. Metal-ceramic restorations were selected because of financial status of this patient. Restoring of a tooth that has undergone crown lengthening is commonly performed in 4 to 6 weeks after the surgical procedure but a clinical study has demonstrated that the biologic width and the position of the free margin of gingiva exhibited no change at 3 to 6 months after surgery [36]. This patient was restored with provisional restoration after 6 weeks of crown-lengthening procedure, and the final restorations were made after 3 months of periodontal surgery.

## 6. Conclusion

AI is a developmental disorder that can result in reduced oral-health quality of life and causes psychological problems. Thus, these patients need extensive treatment. Coordinated orthodontic, periodontal, prosthodontics, and restorative treatments, with careful consideration of patient expectations, requests, and financial status, were critical for a successful outcome and patient satisfaction. Early treatment of patient with AI disorder can prevent progressive damage of dentition and the psychological impact of this condition.
